# A Systematic, Open-Science Framework for Quantification of Cell-Types in Mouse Brain Sections Using Fluorescence Microscopy

**DOI:** 10.3389/fnana.2021.722443

**Published:** 2021-12-06

**Authors:** Juan C. Sanchez-Arias, Micaël Carrier, Simona D. Frederiksen, Olga Shevtsova, Chloe McKee, Emma van der Slagt, Elisa Gonçalves de Andrade, Hai Lam Nguyen, Penelope A. Young, Marie-Ève Tremblay, Leigh Anne Swayne

**Affiliations:** ^1^Division of Medical Sciences, University of Victoria, Victoria, BC, Canada; ^2^Axe Neurosciences, Centre de Recherche du CHU de Québec, Université de Laval, Québec City, QC, Canada; ^3^Department of Neurology and Neurosurgery, McGill University, Montréal, QC, Canada; ^4^Department of Molecular Medicine, Université de Laval, Québec City, QC, Canada; ^5^Department of Biochemistry and Molecular Biology, University of British Columbia, Vancouver, BC, Canada; ^6^Djavad Mowafaghian Centre for Brain Health, University of British Columbia, Vancouver, BC, Canada; ^7^Department of Cellular and Physiological Sciences, University of British Columbia, Vancouver, BC, Canada; ^8^Department of Neurology and Neurosurgery, Centre for Research in Neuroscience, Brain Repair and Integrative Neuroscience Program, Research Institute of the McGill University Health Centre, Montreal General Hospital, Montreal, QC, Canada

**Keywords:** open science, fluorescence microscopy, image analysis, mouse brain, reproducibility, experimental design, neuroscience

## Abstract

The ever-expanding availability and evolution of microscopy tools has enabled ground-breaking discoveries in neurobiology, particularly with respect to the analysis of cell-type density and distribution. Widespread implementation of many of the elegant image processing tools available continues to be impeded by the lack of complete workflows that span from experimental design, labeling techniques, and analysis workflows, to statistical methods and data presentation. Additionally, it is important to consider open science principles (e.g., open-source software and tools, user-friendliness, simplicity, and accessibility). In the present methodological article, we provide a compendium of resources and a FIJI-ImageJ-based workflow aimed at improving the quantification of cell density in mouse brain samples using semi-automated open-science-based methods. Our proposed framework spans from principles and best practices of experimental design, histological and immunofluorescence staining, and microscopy imaging to recommendations for statistical analysis and data presentation. To validate our approach, we quantified neuronal density in the mouse barrel cortex using antibodies against pan-neuronal and interneuron markers. This framework is intended to be simple and yet flexible, such that it can be adapted to suit distinct project needs. The guidelines, tips, and proposed methodology outlined here, will support researchers of wide-ranging experience levels and areas of focus in neuroscience research.

## Introduction

Historically, neuroscientists have used microscopes to identify different cell types and determine their distribution in the nervous system. Analysis of cell types provides important information on neurodevelopmental processes and neurological disease states. There is a narrow range of acceptable variance in cell type density and distribution, outside of which is associated with neurological and neuropsychiatric disorders (Stoner et al., 2014; Bernard et al., 2017; DeTure and Dickson, 2019; Briscoe and Marín, 2020). Studies of this kind stemmed from the influential work of neuroscientists such as Ramon y Cajal, Golgi, del Rio-Hortega, and others, who provided the initial morphological descriptions of neurons and glial cells (Glickstein, 2006; Garcia-Lopez et al., 2010; Sierra et al., 2016). Since its inception, microscopic examination of the brain has evolved markedly. Transformative advances in immunostaining techniques, *in situ* omics technologies, cell-type specific transgenic reporter models, and microscope capabilities (Wilt et al., 2009; Daigle et al., 2018; Yuste et al., 2020) have not only expanded our understanding of the cellular composition of the brain in health and disease, but have also promoted the creation of highly diverse tools and approaches to analyze these types of data. As a result, there is significant variability in the processes used to generate data (e.g., density and distribution) on cell types in the brain, leading to significant challenges when it comes to integrating, interpreting, and reproducing published data (Martone et al., 2004; Geschwind and Konopka, 2009; discussed in Akil et al., 2011). To help mitigate these challenges, various groups have encouraged the adoption of open science frameworks rooted in the Findable, Accessible, Interoperable and Re-usable (FAIR) Data Principles (Wilkinson et al., 2016). Platforms and initiatives supporting open science dataset production and analysis, such as, WholeBrain, BrainGlobe, and Atlas Based Analysis (Niedworok et al., 2016; Fürth et al., 2018; Bourgeois et al., 2021; Tyson et al., 2021), have tremendous potential, yet are still somewhat lacking in accessibility (computing equipment and required expertise). With the on-going push from the neuroscience community to standardize the design, interpretation, and analysis of research studies, there is an unmet need for open science frameworks for experimental design and analysis of fluorescence microscopy images (Bernard, 2019; Picciotto, 2020).

**FIGURE 1:**
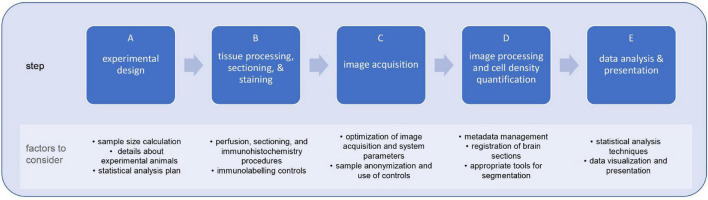
Steps and limiting factors involved in planning and executing a research study on cell-type quantification in brain sections. Research studies for the quantification of cell-types in the mouse brain are sequential multi-step processes (A–E), each with their own limiting factors. By formulating this type of studies within a systematic framework, researchers can mitigate such limiting factors and, consequently, increase the reliability, reproducibility, and usability of study outcomes.

To this end, we provide a framework for neuroscientists of varying expertise and area of research focus rooted in open-science principles and user-friendly resources. We highlight the standard steps and consideration of a research study investigating cell-types in mouse brain, including: statistical power and sample size estimation (Button et al., 2013; Picciotto, 2020), selection of control groups (Tye, 2018; discussed in Picciotto, 2019), sex and genetic background (Kiselycznyk and Holmes, 2011; Vanden Berghe et al., 2015; McCarthy et al., 2017; Luo et al., 2020), image acquisition and processing (Aaron and Chew, 2021; Heddleston et al., 2021), and data visualization and statistical analysis (Calin-Jageman and Cumming, 2019) (Figure 1). Moreover, we provide a step-by-step FIJI-ImageJ image analysis workflow to quantify cell density in the mouse brain sections. Altogether, this work aims to serve as a ‘starter guide’ for facilitating systematic and programmatic analysis approaches and promoting the benefits of open science frameworks in neuroanatomical cell type quantification in mouse brain.

## Methods and Results

In the following section and subsections, we will outline and discuss important aspects that aid in systematic and open-science-based design, execution, and reporting of quantification of cell types in mouse brain sections. Within this, we include a FIJI-ImageJ-based workflow to register brain section images, segment region of interest and quantify cell types in them.

### Experimental Design

A critical, often overlooked, first step in a study aimed at quantification of cell-types in the mouse brain, is detailed experimental design development. Experimental design is not only the planning of experimental procedures, but also the consideration of study design (e.g., groups to compare, selection of control groups, and determination and balanced allocation of experimental units), sample size determination (e.g., number of experimental units per group, *a priori* sample size calculations), strategies to mitigate bias (e.g., objective inclusion/exclusion criteria, randomization, identity concealment), details about experimental animals (e.g., species, strains, and substrains, sex, age or developmental stage), experimental procedures (e.g., description of the intervention, timing, location, and rationale), and the statistical analysis plan, expected outcomes, and delivery of results (e.g., presentation of summary and descriptive statistics, measurements of variability, and effect size with confidence intervals when applicable). The steps mentioned above are included in the “Essential 10” item list developed as part of the Animal Research: Reporting of *in vivo* Experiments (ARRIVE) guidelines (du Sert et al., 2020a,b). These guidelines were designed to help researchers identify key reporting information for animal research, with the aim of increasing reproducibility. Moreover, a well-designed experimental animal study can reduce the number of animals required, and refining experimental procedures can minimize their adverse effects (Festing and Altman, 2002). Studies on mouse brain cell-type densities should include balanced experimental groups with adequate numbers to detect differences between such groups, whilst acknowledging and accounting for strain issues, and sex differences.

In terms of the calculations involved in determining animal numbers within experimental groups, we support the recommendations given in recent editorials in The Journal of Neuroscience and eNeuro (Bernard, 2019; Picciotto, 2019, 2020), for the integration of pilot experiments and power analysis to calculate sample sizes, with the goal of increasing statistical power (defined as 1 – β, where β is the probability of a type II error or, in other words, the probability of accepting a null hypothesis that is actually false – i.e., the false-negative rate–) and consequently increasing the reliability of observed results (Picciotto, 2020). Fortunately, estimating sample size by power analysis is possible with openly available online tools^1^,^2^ or free statistical software (e.g., R and the package “*pwr*”) but require researchers to determine the effect size via pilot studies or previous reports. An alternative can be found in the “resource equation” (Equation 1). The resource equation is based on the notion that a good estimate of error requires at least 10 degrees of freedom (defined as experimental units – 1) (Mead, 1988; Mead et al., 2012; Festing, 2018) and becomes particularly useful when an effect size cannot be determined through pilot studies or approximated from previous literature.


(1) E=total⁢number⁢of⁢experimental⁢units-total⁢number⁢of⁢treatment⁢groups⁢(or⁢genotypes)

where the value of E should be between 10 and 20.

Using this approach and considering the relationship between sample size, power, and effect size (Ellis, 2010; Festing, 2018), researchers can obtain a predicted effect size for a given provisional sample size. From here, given that statistical power increases with size and effect size, the sample size can be adjusted until reaching an effect size that is reasonably acceptable or detectable (Supplementary Figure 1) (Ellis, 2010; see Table 1 in Festing, 2018). For example, if researchers plan to detect the differences of a given cell-population between two genotypes (i.e., 2 groups), they will require between 12 and 22 total animals (i.e., 6–11 animals per genotype). The same researcher could use a plot (Supplementary Figure 1) or table showing the relationship between sample size and standardized effect size to learn the statistical power in the example above.

In terms of proper consideration of the impact of mouse strain (and substrain) genetics and sex, it is important to recognize and mitigate the genetic and phenotypic variation present across and within mouse strains, as reviewed by Chen and Flint (2021) and Shansky and Murphy (2021). For example, it is well-established that C57BL/6, an inbred strain of mice frequently used as wildtype controls, is phenotypically distinct between substrains (e.g., C57BL/6J, C57BL/6N, C57BL/6NTac, and C57BL/6NCrl), highlighting the need to carefully check the mouse transgenic strains (and substrains) in experimental group allocation (Crawley et al., 1997; Beck et al., 2000; Matsuo et al., 2010; Kiselycznyk and Holmes, 2011; Zurita et al., 2011; Kang et al., 2015). These groups must be balanced (i.e., equal numbers of experimental animals per group) and include both sexes whenever possible (Beery and Zucker, 2011; McCarthy et al., 2017; Mamlouk et al., 2020; Woitowich et al., 2020; Shansky and Murphy, 2021). We recommend that researchers refer to the Mouse Genome Informatics website^3^ for precise information (e.g., nomenclature, strain-specific single nucleotide polymorphism, phenotypes, gene expression, and Cre-recombinase activity), especially ‘‘*Me-PaMuFind-It*’’^4^ to identify potential 129S-derived mouse strain passenger mutations (particularly if the strain has not been backcrossed substantially). Consistent use of these resources is critical to prevent and mitigate possible confounds secondary to germline recombination (Luo et al., 2020) and passenger mutations (Vanden Berghe et al., 2015).

With respect to statistical analysis, we support the call for a shift from hypothesis-testing-based statistics toward the more widespread adoption of estimation statistics (Bernard, 2019; Calin-Jageman and Cumming, 2019). Null hypothesis significance testing has been the main framework used by researchers to make predictions of a population from observations of representative sample (McDonald, 2014). Within this framework, observations are measured, summarized, and a *p*-value is estimated to declare whether there are “statistically significant” differences based on a *p*-value threshold established, somewhat arbitrarily by RA Fisher (1956), at 0.05 (Gelman and Stern, 2006). On the other hand, estimation statistics emphasizes effect sizes and measures of uncertainty, providing a quantitative assessment of observed differences (and their variability) rather than a binary outcome (i.e., statistically significant *vs.* not statistically significant) (Calin-Jageman and Cumming, 2019; Wasserstein et al., 2019). While biomedical animal research has heavily relied on null hypothesis significance testing (Gigerenzer, 2018), strong cases have been made that estimation statistics approaches improve interpretation of results (by presenting effect sizes, confidence intervals, and data distribution), tackling overconfident interpretations based on a *p*-value threshold, and consequently, improving statistical inference (Calin-Jageman and Cumming, 2019; Bernard, 2021). Moreover, establishing a statistical plan before data is collected and that is complemented by the use of estimation statistics can contribute to prevent malpractices such as “harking” or “*p*-hacking,” which refer to hypothesizing after the data is known, and selecting statistical tests according to the outcome of a study, resulting in ill-bias and interpretation errors when reporting the outcomes of a study (Kerr, 1998; Head et al., 2015; Picciotto, 2018, 2020; Bernard, 2019). Another editorial called for the adoption of analyses using estimation statistics with the goal of improving the interpretation of results (by presenting effect sizes, confidence intervals, and data distribution), tackling overconfident interpretations based on a *p*-value threshold, and consequently, improving statistical inference (Bernard, 2019, 2021; Calin-Jageman and Cumming, 2019).

### Tissue Processing, Sectioning, and Staining

Accurate quantification of cell types in mouse brain sections requires consistent and optimal tissue preparation. Preserving the integrity of the cellular and extracellular components is often accomplished by perfusing an animal with a buffered solution followed by a fixative solution (Gage et al., 2012). Fixatives are typically diluted in a buffered physiological solution (for example, 4% paraformaldehyde diluted in phosphate buffered saline) and the selection of a specific fixative should be tailored to subsequent histological processing and intended applications (Gage et al., 2012; Zhang and Xiong, 2014). Similarly, controlling the flow (by using a peristaltic pump or a simple gravity feed) and temperature of a fixative and assigning tissue extraction and dissection to trained personnel are important aspects that contribute to consistency across samples (Paul et al., 2008). For example, delayed perfusion of fixative (∼5–8 min delay between the initial cut of the diaphragm and the time of visible clear paraformaldehyde outflow) can alter the distribution and detectability of proteins, resulting in inaccurate quantification when compared to fully perfusing an animal within 100 s from cutting the diaphragm (Tao-Cheng et al., 2007). Likewise, whole body perfusion using 4% paraformaldehyde without perfusing first a buffered solution can lead to aberrant vacuolation of neuronal somas (Leonard et al., 2016). The type of fixative used, and its concentration depend on downstream applications. For example, synaptic proteins are sensitive to paraformaldehyde perfusion (Wallrafen et al., 2019), lipophilic dyes required lower concentrations of paraformaldehyde (1.5% or 2%) (Li et al., 2008; Rasia-Filho et al., 2010; Staffend and Meisel, 2011). Glyoxal is known to nicely preserve cellular structures such as actin filaments and microtubules (Jones, 1969). Glutaraldehyde produces higher autofluorescence and irreversible cross-links of proteins (whereas paraformaldehyde does not), acrolein cross-links at higher rate than paraformaldehyde and rapidly penetrates tissues (Jones, 1969), while methanol leads to a fast fixation with loss of organelles (Spector and Goldman, 2006; Richter et al., 2018; Celikkan et al., 2019; Yao et al., 2021). Furthermore, certain paradigms use drop fixation in place of perfusion (limited to 1 mm/per hour of penetration) which causes differences in glial cell morphology (Cǎtǎlin et al., 2017). Because the effects of the above sample preparation profoundly impact downstream analyses (Pereira et al., 2019), we recommend researchers to adopt a quality control checklist. In Supplementary Table 1 (“Tissue processing”), we present an example of such list and provide recommendations. Needlessly to say, the information within the checklist also serves as a useful record of important items to include when reporting a study.

Similarly, it is important to provide details of procedural steps related to sectioning, such as equipment type (e.g., vibratome, cryostat, or freezing microtome), orientation and thickness of sections, embedding medium, use of cryoprotective and preservative reagents, and if sections have been air-dried, all which can impact the quality of immunohistochemistry labeling and morphological metrics (Bacallao et al., 2006; Spector and Goldman, 2006; Estrada et al., 2017). Additionally, details of immunohistochemistry procedures such as use of permeabilization, blocking, labeling, and mounting agents, and their respective incubation/application time should all be included within the ‘‘Methods And Results’’ section. Researchers should use research identifiers (RRID^5^) for all reagents, particularly dyes and antibodies, and note subsequent manipulations (e.g., adding glycerol to antibodies, diluting dyes in methanol, sonication, etc.) in their Methods. Lastly, the researchers are recommended to include information on immunolabeling controls, such as antigen positive and negative controls, background controls, and reagent controls (Spector and Goldman, 2006; Lee and Kitaoka, 2018; Jonkman et al., 2020).

### Image Acquisition: Brief Overview of Foundations and Best Practices

A solid theoretical and practical foundation in light microscopy is vital to carry on reproducible and replicable microscopy-based studies (Thorn, 2016; Lee and Kitaoka, 2018). Numerous factors, if poorly understood or not properly considered, can result in inadequate image quality and variability in measurable outcomes (Aaron and Chew, 2021; Heddleston et al., 2021). For example, limited understanding of the diffraction limit in optical systems and the relationship between an objective numerical aperture (*NA* = n*sin⁡θ, where *n* is the refraction index of the medium, θ is the half-angle of the cone of light which can be collected by the objective lens, and *NA* is numerical aperture) and resolution ($r\,e\,s\,o\,l\,u\,t\,i\,o\,n=\frac{1.22\,\lambda }{2\,N\,A}$) can result in acquisition of poorly resolved images. This issue can be solved by optimizing image acquisition to follow the *Nyquist sampling* principle, in which (for an optical system) the pixel size should be at least two to three times smaller than the resolvable element to capture at the full resolution of the objective (Pawley, 2006; Thorn, 2016; Jonkman et al., 2020). Free online resources such as iBiology Microscopy Series which hosts lectures, virtual laboratories, and self-assessments offer a comprehensive review of the fundamentals of optics and microscopy^6^, facilitating learning and providing a reference resource to junior and senior researchers. For example, a factor such as brain section thickness plays a critical role in selecting the appropriate type of microscope for a given study: widefield microscopes perform best with sample thickness of 20 μm or less, while spinning-disk and confocal laser-scanning microscopes perform well with a sample thickness between 30 and 50 μm and up to 200 μm, respectively, thanks to their improved resolution in the *z*-axis (depth) (Thorn, 2016). Likewise, recent advances in technology, such as light-sheet microscopy and tissue clearing, can offer excellent performance to quickly image large volumes (Keller and Ahrens, 2015; Susaki and Ueda, 2016; Thorn, 2016); while this technology is not yet widely available and requires intricate sample preparation protocols, it holds terrific potential as engineering and optic development continue to improve its resolution and usability by non-optic specialists (Mano et al., 2018; Albert-Smet et al., 2019; Lu et al., 2019).

Robust microscopy imaging acquisition incorporates mitigation of confirmation bias, through, for example, sample anonymization, allocation concealment, and use of controls. Imaging acquisition parameters should be established using a positive control (e.g., a sample in which the protein of interest is known to be expressed) and be tested on a negative control sample (e.g., a sample from tissue that does not express the protein of interest, or a sample that was not incubated with the labeling reagent). The parameters should allow the researcher to perform image acquisition using as much of the dynamic range of the detector as possible (North, 2006). Imaging parameters should be optimized to prevent oversaturation or undersaturation, as both of these represent loss of information, and therefore data (however, note that it is critical to extract the background before conducting any quantitative image analysis) (Brown, 2007). Fortunately, many modern-day microscopes and their software suites offer options to visualize, in real time, the intensity distribution of a given image using histograms and high/low or range finder look-up table (LUT), but even when using systems that do not offer these options [for example, do-it-yourself microscope systems for research and education (Gibbs et al., 2018; Grier et al., 2018; Flores and Marzullo, 2021)], open source tools such as FIJI/ImageJ can be used to assess these parameters (Schindelin et al., 2012). Once the parameters have been established, these should be kept constant throughout an experiment (North, 2006). Lastly, acquisition parameters should aim to maximize the signal-to-noise ratio, the relationship between actual signal and background signal. While acquisition parameters can significantly influence signal-to-noise ratio, additional aspects such as the sample preparation, selection of high quantum yield labels, type of sensors and their gain, selection of objectives, and environment (e.g., temperature and vibration) can have an effect (Ogama, 2020). Note that increasing the gain of a sensor does not improve signal-to-noise ratio, as it increases the detection of both the actual signal and background. To increase signal-to-noise ratio with a properly prepared sample using optimized fluorescence labels, one can increase the exposure time or use stronger excitation (higher laser power); however, both of these strategies result in increased phototoxicity and photobleaching (Ogama, 2020). Table 1 summarizes some of the factors that influence signal-to-noise ratio.

**TABLE 1:** Overview of factors that affect the signal-to-noise ratio (SNR) in fluorescence imaging.

Factor	Examples	Use	Signal-to-noise	Mitigation	References
**Mouse model**					
Model-specific considerations, e.g., aging, neurodegeneration models	Aged mice.	Experimentation.	Lipofuscin pigment increases with age and autofluoresces.	Photo-bleaching.	Sun and Chakrabartty, 2016; Alegro et al., 2017; Jenvey and Stabel, 2017; Gao et al., 2019
	Neurodegenerative disease transgenic mouse models.		Amyloid deposits autofluoresce.	Adjustment of laser power and detection wavelength.	Dickey et al., 2003; Gao et al., 2019
**Tissue preparation**					
Perfusion/fixation	Examples of perfusion methods include brain-targeted, and dual perfusion.	Blood removal and tissue preservation.	Blood pigments autofluoresce, and this becomes pronounced with prolonged fixation.	Select fixative and perfusion method best suited for experiment and ensure. Steady perfusion flow rate.	Jenvey and Stabel, 2017; Whittington and Wray, 2017; Belhadjhamida et al., 2020
Dissection and sectioning	Manual macrodissection, manual microdissection, and laser microdissection. Brain sections.	Isolation of high-quality samples from a given region of interest.	Tissue damage during dissection can lead to exaggerated cell death/apoptosis, leading to autofluorescence. Section thickness affects antibody penetration. Thicker sections exhibit reduced labeling and increased light scattering.	Establish quality control checks and standardized operating procedures. Match tissue section thickness with the resolvable power of a microscope system.	Spanswick et al., 2009
**Immunohistochemistry**					
Blocking	Normal serum, species-specific serum, bovine serum albumin, gelatin, casein, non-fat dry milk, or biotin.	Used to reduce non-specific antibody binding and labeling.	Use of a blocking agent from the same species in which the primary antibody was raised can lead to reduced secondary antibody binding.	Include a blocking incubation step when using indirect immune fluorescence	Jenvey and Stabel, 2017; Im et al., 2019
Primary antibody	Methods include direct (one-step incubation process) and indirect (two-step incubation process) immunofluorescence.	Binds to a protein/biomolecule of interest to the research project.	Primary antibody cross-reactivity, specificity, affinity and concentration.	Select thoroughly tested primary antibodies with high antibody specificity (tested in knock out tissues) and affinity. Optimize antibody concentration.	Burry, 2011; Im et al., 2019
Secondary antibody	Used for indirect (two-step incubation process) immunofluorescence.	Binds to the primary antibody.	Use of secondary antibodies to the same host species as the sample can result in cross-reactivity with endogenous immunoglobulins. Fluorophore bleaching.	Include secondary antibody controls in your experiments. Select a secondary antibody against the host species of the primary antibody. Optimize antibody concentration.	Burry, 2011; Im et al., 2019
**Microscopy**					
Laser excitation	Varies by manufacturer.	Illuminate (excite) sample.	Increase laser power appropriately, factoring in photobleaching and phototoxicity.		Brown, 2007; Ogama, 2020
Detection	Filter sets and beam splitters. Detectors.	Separate illumination (incident light) from detection (emitted light).	Select appropriate filter sets to mitigate or eliminate crosstalk. Select filter sets that match the emission wavelength of the fluorescence label applied to a sample. Acquire images sequentially to mitigate crosstalk in exchange of acquisition speed. Increases in detection gain increase both the specific and the non-specific “background” signals simultaneously. With laser-scanning, line/frame averaging helps to average noise whilst accumulating signal.		
Objectives	Varies by manufacturer.	Gather reflected light to form images.	Use high NA objectives and match immersion medium and sample mounting medium, when possible.		
Environment	Temperature and humidity. Environment light contamination. Vibration.	NA.	Across replicates, samples should be exposed to consistent ambient light levels, temperature, and humidity during image acquisition. Imaging should (ideally) be performed in a dark room. Test the environment background signal acquired by the detector in the absence of sample. Mount microscope systems on anti-vibration tables.		

Lastly, a key area for improvement in published microscopy-based studies is the reporting of imaging methods. A recent analysis of 240 original research articles published in 8 different journals revealed that imaging methods were only included in approximately 5% (range 2.3–10.2%) of biomedical research papers. Moreover, less than one-fifth of the studies provided sufficient details on imaging methods (Marqués et al., 2020), such as imaging acquisition parameters and the collection of metadata (Linkert et al., 2010; Huisman et al., 2021). Fortunately, various resources exist in the form of reviews that provide great recommendations on how to improve rigor in acquiring, reporting, and analyzing (discussed in following sections) microscopy data (Lee and Kitaoka, 2018; Aaron and Chew, 2021; Heddleston et al., 2021). Table 2 includes a set of recommended image acquisition parameters to be reported to promote reproducibility and replicability.

**TABLE 2:** Methodological image acquisition parameters to report in scientific publications*.

Microscope component/acquisition property	Parameter
Microscope	Manufacturer. Upright or inverted. Microscope operating software (version and maker).
Light source (Lasers)	Source type (gas, semiconductor, and crystal). Manufacturer and model. Wavelength. Power.
Optics and stage	Dichroic mirror or beam splitter information (wavelength and manufacturer). Excitation/emission filters (manufacturer and wavelength). For confocal microscopes: pinhole size. Stage motor, incubation chamber, and custom hardware.
Objectives	Manufacturer. Magnification. Numerical aperture (NA). Type of optical aberration correction (Achromat, Plan Achromat, Fluorite, Plan Fluorite, and Plan Apochromat). Medium and refractive index.
Detection	Detector type and manufacturer. Exposure/pixel dwell time. Gain. Offset. Binning (if applicable). Line/frame averaging, accumulation (for laser-scanning confocal microscopes).
Image size and acquisition	*x*-, *y*-pixel size, *z* step size, and *t* interval for time-lapse experiments. Total image size (metric and pixels). If using a multi-channel compatible system, report whether channels were acquired simultaneously or sequentially (in line, in frame, in stack). Bit depth (8, 12, or 16 bits).
Image processing	Signal enhancement: details about background subtraction (kernel size and shape), denoising (kernel size and shape, noise sigma and smoothing value), filtering (frequency cut-off values), deconvolution (estimated PSF, number of itierations). If using intensity threshold, report automated method or used values. Note that manual selection of values is prone to user-bias. Segmentation process, including binary operations, size exclusion, shape parameters.

*From session to session it is recommended to maintain consistent focus, tissue depth, light intensity, and detection settings (Lee and Kitaoka, 2018; Aaron and Chew, 2021; Heddleston et al., 2021).

### Framework for Image Processing and Cell Density Quantification in Brain Sections

To increase data reproducibility, various institutions (e.g., funding body, journal requirements, and international collaboration consortiums) have promoted an increased use of quantification in fluorescence microscopy studies (Aaron and Chew, 2021). Earlier neuro-morphometric studies relied on stereology as a method to mitigate variability and inconsistencies (Haug, 1986; Zhao and van Praag, 2020) within small regions; however, newer advances and initiatives permit the quantification of cell density across large regions and even the whole brain at an unprecedented speed. In this regard, neuroscience-specific initiatives, such as the Brain Initiative’s functional connectome project, the Allen Brain Institute Reference Atlas, WholeBrain, and BrainGlobe provide excellent resources and tools (Fürth et al., 2018; Anderson et al., 2021; Claudi et al., 2021). However, barriers such as user-friendliness, proficiency in programming languages, and other inherent restrictions – for example, the requirement for large whole brain section images – still limit the wide application of many of these tools. While image analysis tools continue to evolve and become flexible to a wider range of researchers’ experimental needs and resources, FIJI-ImageJ continues to be the most commonly used image analysis suite, including for the analysis of cell population quantification in brain sections (Schindelin et al., 2012; Bourgeois et al., 2021). For example, Bourgeois et al. (2021) recently proposed a FIJI-ImageJ-based semi-automated atlas-based workflow to obtain cell counts from mouse brain sections using the Paxinos and Watson rat brain atlas for anatomical reference (Paxinos and Watson, 2006), resulting in decreased inter-observer variability and a faster generation of data. However, this workflow is limited by the use of the adult rat brain anatomical reference to register mouse anterior–posterior coordinates and relies on manual tracing to outline regions of interests within cortical (i.e., cortical layers) and subcortical regions, which is prone to technical and human error. With the above in mind, we have developed a simple, adaptable, semi-automated framework prioritizing ease of use, metadata and data management, image quality control, registration to a unified mouse atlas, expandability, and implementation of a machine-learning tool for feature extraction and segmentation (“*StarDist*”). In the following sections we will describe the steps necessary to use our FIJI-ImageJ based framework to quantify cell density in mouse brain sections.

#### Loading Image Data

To begin using our image processing and analysis workflow the user first runs a file management script (step 1^7^) and loads their image files into FIJI-ImageJ. This script takes image series stored in a proprietary file format (e.g., “.lif,” “.czi,” and “.oir,” for Leica Microsystems, Zeis Microscopy, and Olympus Microscopy, respectively) and exports these image series in a “.tif” (a file format that complies to FAIR specifications, (Wilkinson et al., 2016; Swedlow et al., 2021). Files are organized into an “output” folder, comprising subdirectories for each image series and subsequent associated files. Multi-channel image series containing z-sections are automatically split into individual channels along with the generation of their maximum intensity projections, including a channel-merged image file. For each image channel, a pixel intensity frequency table is generated and stored (as a “.csv” file) within its respective image series subdirectory. This pixel intensity frequency table can help users assess the distribution of pixels within the image and detect images with over-representation of over-saturated or very dark pixels that might not be fully suitable for further image processing (Brown, 2007). Moreover, this table can be further analyzed using online tools such as ‘‘*ggPlotteR*’’^8^ or “*PlotsofData*” (Postma and Goedhart, 2019). Lastly, the macro also calls a function to extract and store metadata for each opened image data series as “.csv” files (Linkert et al., 2010; Huisman et al., 2021).

#### Registering Brain Sections

The mouse brain section registration in our proposed semi-automated image analysis framework is based on a freely available enhanced and unified atlas generated by Dr. Yongsoo Kim’s laboratory at Penn State University^9^ (Chon et al., 2019), which merges the coordinates and labels from the mouse stereotactic atlas by Franklin and Paxinos (2008) with those from the Common Coordinate Framework developed with the Allen Brain Institute Mouse Reference Atlas (Sunkin et al., 2012; Kuan et al., 2015). This unified atlas was refined using magnetic resonance imaging in male and female Ai14 reporter mice at 2–3 months old. Using a unified atlas for image registration is expected to facilitate data integration and cross-analysis of datasets with other open-science data resources (e.g., transcriptomic and electrophysiologic data) generated by institutions (e.g., the Allen Brian Institute) and researchers, while preserving the familiarity of the Franklin and Paxinos coordinate system.

**FIGURE 2:**
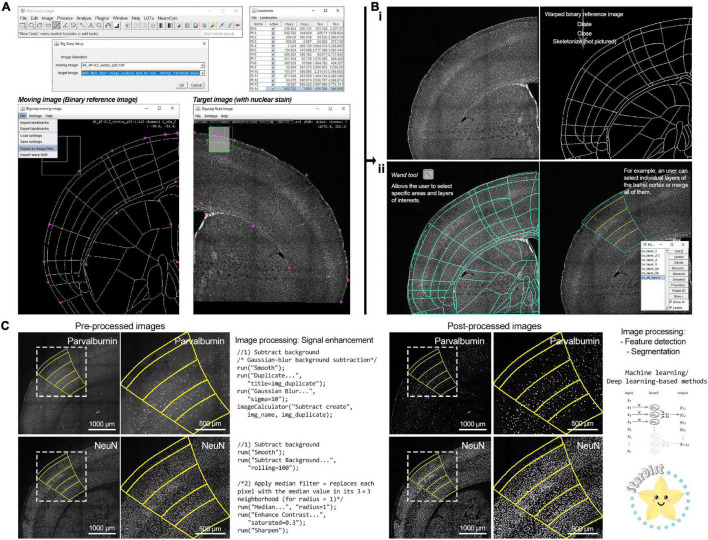
A FIJI-ImageJ based workflow for registration and image processing of mouse brain sections. **(A)** Brain section images can be registered to a unified atlas using the FIJI-Image-J plugin “*Big Warp*.” Using a nuclear stain (such as Hoechst) facilitates the placement of anatomical landmarks to adjust the reference atlas outline to the brain section image. **(B) (i)** The “warped” reference atlas outline can be converted into a binary mask. **(ii)** By binarizing the reference atlas outline, the user has the ability to select specific anatomical regions of interest (ROI) using the “*Wand tool.*” Individual ROIs can be merged in the FIJI-ImageJ “*ROI Manager*” with the operator “*OR*” to select larger brain areas. **(C)** Fluorescence microscopy images often require signal enhancement processing (targeted primarily to reducing background noise) to make them suitable for application of feature extraction and segmentation algorithms. Uniform and consistent signal enhancement processing can be achieved through scripts. Signal-enhanced images can be further processed with machine learning-based tools, such as “*StarDist*.” The “*StarDist*” logo was used with permission from copyright holder Dr. Martin Weigert. The convolutional neural network diagram is published under the Creative Commons Attribution-Share Alike 4.0 International license (Vicente Oyanedel M., CC BY-SA 4.0; URL: https://commons.wikimedia.org/wiki/File:1D_Convolution.png).

Similar to Bourgeois et al. (2021), upon matching an image series with an anteroposterior coordinate, the user can download the corresponding image from the unified atlas generated by Yongsoo Kim’s laboratory at Penn State University [see Text Footnote 9; (Chon et al., 2019)] and process the included .ai file with a vector graphic software, such as Inkscape (free open source software by The Inkscape Project) or Adobe Illustrator (licensed by Adobe Inc.) to export the included outline as a ‘‘.png,’’ a compatible file format for FIJI-ImageJ. Using our second macro (step 2^10^) the user can upload and deform (“warp”) the “.png” file containing the outline following the imaged brain section with FIJI’s ready-to-use plugin “*BigWarp*” (Bogovic et al., 2016), and binarize the files within FIJI-ImageJ (Figure 2A). For this process, with the two images side-by-side (we recommend using a strong nuclear stain, such as Hoechst for this step), the user can place landmarks (15–20; landmarks can be managed with the “*Landmark Analyzer*”) using characteristic anatomical features such as the lateral ventricles, corpus callosum, and boundaries of the dorsolateral cortical surface (Figures 2A,B). To note, the warping process can create 1–5-pixel gaps on the outline, which requires the addition of a border and small adjustments using FIJI-ImageJ’s “*Brush tool*” to close such gaps and prepare the “warped” image for further processing. Once this step is complete, the user can use FIJI’s “*Wand tool*” to select their target region of interest. For example, a user can select individual layers within the barrel cortex and transfer them to FIJI’s “*ROI Manager*” or create a full depth cortical selection of the barrel field by selecting and combining all layer regions of interests using FIJI’s “*OR*” function (Figure 2B, bottom half). The area of the selected region of interest is then calculated and stored as a “.csv” file.

#### Segmentation and Quantification

**FIGURE 3:**
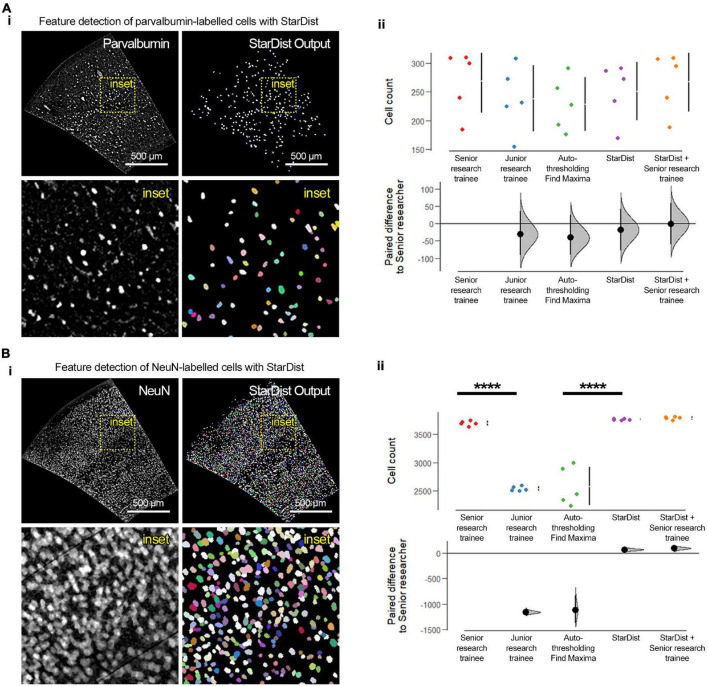
Feature extraction, segmentation, and quantification using “*StarDist*” and other conventional and manual approaches. **(A) (i)** Representative fluorescence micrographs of the barrel cortex with parvalbumin-labeled cells and “*StarDist*” color-coded annotating output of segmented objects. **(ii)** “*StarDist*” was as accurate as a senior research trainee in detecting the number of parvalbumin-labeled cells. Note the similar distribution of the data points between “*StarDist*,” the manual quantification by a senior research trainee, and the two methods combined (“*StarDist*,” mean: 251 ± 50.6 cells; senior research trainee, mean: 268 ± 55.1 cells; “*StarDist*” + senior research trainee: 267.8 ± 52.71 cells; mean ± standard deviation; *p* = 0.694; *d* = –17.8 [95CI –77.8; 42.8]; *d* = –1 [95CI –60.8; 60.4]). **(B) (i)** Representative fluorescence micrographs of the barrel cortex with NeuN-labeled cells and “*StarDist*” color-coded annotating output of segmented objects. **(ii)** As expected, using labeling with NeuN results in images with high cellular density, which are significantly challenging for inexperience research trainees and conventional thresholding approaches (*p* < 0.0001). “*StarDist*” significantly outperformed the autothresholding and “*Find Maxima*”-based conventional approaches in detecting, segmenting, and quantifying labeled cells (“*StarDist*,” mean: 3765.8 ± 13.3 cells; autothresholding /“*Find Maxima*,” mean: 2580.4 ± 339.8 cells; *p* < 0.0001; *d* = 1190 [95CI 907; 1440]). “*StarDist*” performance was similar to that of the manual quantification by a senior research trainee or the two combined, albeit with lower variability (senior research trainee, mean: 3698.6 ± 40.1 cells; “*StarDist*” + senior research trainee, mean: 3789 ± 25.7; *p* = 0.9570; *d* = –17.8 [95CI –77.8; 42.8] and *d* = –1 [95CI –60.8; 60.4], respectively). *N* = 5 randomly selected barrel cortex micrographs; “****”*p* < 0.0001. For experimental details regarding the animals used and image acquisition and processing parameters see Supplementary Methods.

Once a region of interest has been selected, automated counts of labeled cells can be obtained using the included *ImageJ language macro scripts* (step 3^11^,^12^) (Figures 2C, 3). The key image processing steps in order to obtain cell counts are feature extraction and image segmentation. In the digital image processing and computer vision fields, feature extraction refers to the group of image processing operations that detect edges, corner, and segmentation refers to the separation and labeling of objects based on their signal intensity relative to the background and features, breaking the image into smaller fragments and facilitating image interpretation (Nixon and Aguado, 2019). Prior to these processing steps, images must be prepared by subtracting their background and denoising them, leading to an enhancement of the acquired signal (Sternberg, 1983; Nixon and Aguado, 2019). For signal enhancement of images with cells labeled with the neuronal nuclear protein [NeuN; also known as RNA binding protein fox-1 homolog 3 (Wolf et al., 1996; Kim et al., 2009; Duan et al., 2016)] we use FIJI-ImageJ’s “*Subtract Background*” function, while the signal on images with parvalbumin-labeled cells was enhanced by smoothing the image (by replacing each pixel with the average of its 3× neighborhood) and subtracting its Gaussian-blurred duplicate from it (sigma = 10) with the “*Image Calculator*” tool for Figure 2C. Traditionally, feature detection and segmentation workflows for the analysis of cell density in fluorescence microscopy has been based on thresholding (selection of a minimum intensity value to binarize the image) and watershed segmentation (for example, using the auto-thresholding method “*Moments*” alongside watershed segmentation and size/shape filtering for parvalbumin-positive cells and “*Find Maxima*” for NeuN-positive cells; see Supplementary Methods for more details) to identify cells (Sezgin and Sankur, 2004; Aaron and Chew, 2021; Bourgeois et al., 2021). While this approach is not resource intensive and relatively simple, it is prone to significant performance variability, leading to inconsistencies across images (Sezgin and Taşaltín, 2000; Sezgin and Sankur, 2004). To overcome this issue, researchers have developed machine learning tools using deep learning of artificial neural network, which in addition to significant improvements in performance, accuracy, and speed, are adaptable to virtually any dataset by training artificial neural networks (Moen et al., 2019; Meijering, 2020; Hallou et al., 2021; Stringer et al., 2021). While implementing these approaches often requires specialized personnel and high-performance computing equipment, the computing efficiency is being optimized work on regular consumer-graded computing equipment (Moen et al., 2019; Meijering, 2020; Tyson et al., 2021; von Chamier et al., 2021) and there are novel developments in user-friendly implementations (Gómez-de-Mariscal et al., 2021; Lucas et al., 2021; von Chamier et al., 2021). For our FIJI-ImageJ-based workflow, we decided to use “*StarDist*,” a Python implementation for the detection of star-convex objects that uses machine learning and is available as a ready-to-use and user-friendly plugin for FIJI-ImageJ (Schmidt et al., 2018; Weigert et al., 2020). While “*StarDist*” was originally designed to detect cell nuclei in fluorescence microscopy images via a convolutional neural network that approximates nuclei shape with star-convex polygons, “*StarDist*” base-and-ready-to-use models perform well at detecting cell bodies for various fluorescence and brightfield microscopy applications, in particular in challenging cases such as images with high cellular density (Schmidt et al., 2018; Weigert et al., 2020). “*StarDist*” produces two outputs: a 16-bit image containing a color-coded annotation of the segmented objects and the corresponding ROIs for each segmented object (directed to the “*ROI Manager*”). Following the application of “*StarDist*,” our framework exports the segmented objects ROIs, their area, and total object count to a “.csv” files into each image series respective subdirectory. From here, users can perform further downstream analysis with open source statistical software tools such as R and RStudio (R Core Team, 2021) or online R-based and Python-based tools such as “ggPlotteR” (see Text Footnote 8), “PlotsofData” (Postma and Goedhart, 2019), “SuperPlotsofData” (Goedhart, 2021), and “DABEST” (Ho et al., 2019); the latter two are further discussed in the following section.

To validate this workflow, we compared the performance of “*StarDist*” with that of a conventional thresholding and watershed segmentation, manual counting by a junior research trainee (<1 year of experience), and manual counting by a senior research trainee (>5 years of experience) using 10 randomly selected micrographs of the barrel cortex (anteroposterior coordinate +0.2 mm; 5 images with parvalbumin labeling, and 5 images with NeuN labeling) acquired from 30 μm brain sections obtained from C57BL/6J male and female 1-month old mice (Figure 3). The conventional approach resulted in inaccurate and inconsistent detection and quantification of cells. Moreover, this exercise revealed the prowess of “*StarDist*” to detect and segment cells consistently and accurately, even in images with high cellular density such as those containing NeuN-labeled cells (*p* < 0.0001). “*StarDist*” alone, showed a very small error rate of ∼0.8%, calculated based on the *a posteriori* inspection and verification of “*StarDist*” output by a senior researcher (*p* = 0.9991). In the case of images labeled with parvalbumin, no clear difference was observed between analysis methods; however, “*StarDist*” output maintained an error rate of 7%. The inclusion of “*StarDist*” as the feature extraction/segmentation tool within our framework is twofold: first, it significantly speeds up the analysis while maintaining very low error rates and an easy-to-use interactive user interface that allows to tweak the parameters of the object segmentation algorithm to refine it. Second, “*StarDist*” also provides the flexibility to train its convolutional neural network with additional datasets in case the included machine learning models do not accurately detect the cells or structures of interest in a given set of images; albeit this task requires relative expertise in machine learning and its applications for bio-image analysis. While we have developed and validated this framework using coronal sections from 1-month old C57BL/6J mouse brains (postnatal day age 30–33), brain sections from younger or older animals should be still compatible with our framework, as long as brain sections micrographs contain enough anatomical landmarks can be recognized and adjusted using the described plugins.

### Data Analysis and Presentation: Implementing Recent Paradigm-Shifting Advances

The final steps of data analysis, visualization, and sharing and reporting can arguably be the most exciting aspects of scientific discovery. Most biomedical sciences disciplines rely heavily on null hypothesis statistical testing. As a result, *p*-value significance cut-offs have become a binary arbiter of biological importance (Halsey, 2019; Ho et al., 2019). It is important to understand that the *p*-value is an error threshold (Wasserstein et al., 2019), and while these values can identify differences between two groups, they provide no insight into the amplitude of difference. In other words, though a statistical test comparing two means can result in a statistically significant *p*-value (<0.05), the difference between those means may not have any biological relevance [an excellent example in neuroscience is provided in Calin-Jageman and Cumming (2019)]. While *p*-values cannot resolve the extent of observable difference, a measure that can provide this insight is effect size, obtained with estimation-based statistical analysis (Ho et al., 2019). For this reason, we strongly advocate to complement null hypothesis statistical testing with estimation-based statistical analysis, available in open-source web-packages such as “DABEST” (“data analysis with bootstrap-coupled estimation”) (Ho et al., 2019). The advantage of estimation statistics is to report quantitative data and eliminate statistical uncertainty by providing the potential for error, reported as confidence interval estimates (Calin-Jageman and Cumming, 2019), enabling the comparison of results from different studies/contexts (Bernard, 2019; Calin-Jageman and Cumming, 2019; Ho et al., 2019). Lastly, because statistical analysis goes hand in hand with appropriate data visualization and presentation, we recommend plotting data according to the recommendations outlined by Lord et al. (2020) in their “SuperPlots” paper and estimation graphics such as Gardner-Altman and Cummings estimation plots (Ho et al., 2019). The key points can be summarized as follows: (1) display all data points, including all technical and biological replicates; (2) avoid presenting data using bars or boxplots; (3) always display a measure of variability, such as the standard deviation or confidence intervals; and lastly (4) add the results of the statistical test used. Both DABEST and “SuperPlots” are suitable tools to communicate data variability and reproducibility. These plots can be easily generated through free and open-source web tools (Ho et al., 2019; Goedhart, 2021), thereby dismantling barriers to reproducible data visualization and analysis.

## Discussion

**FIGURE 4:**
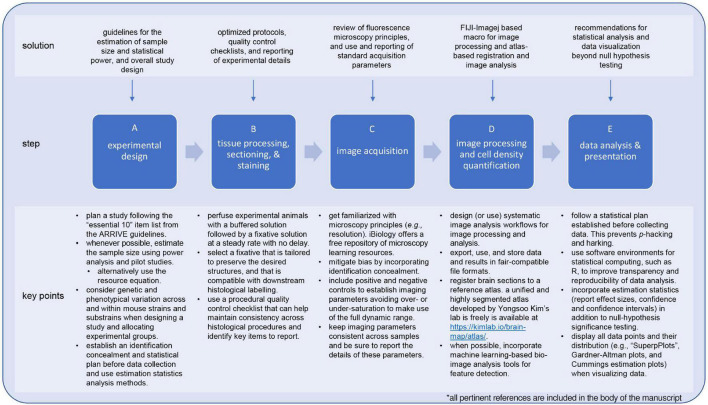
Solutions and recommendations for the implementation of systematic and open science framework to studies for quantifying cell-types in the mouse brain. Familiarization with experimental design concepts and principles helps to identify sources of bias early on and establish plans to mitigate these, resulting in conducting research in an efficient and reliable way. For example, using the ARRIVE guidelines to design an experimental plan not only provides a procedural a structure but also a reference to identify critical items to report on a study (A). Likewise, be acquainted with research methods and equipment is paramount to keep consistency across processed samples (B,C). Using quality control checklist for these steps can facilitate achieving this goal while at the same time provide a reference when it comes to reporting the study. When it comes to process images and extracting data, using workflows based on user-friendly and open-source tools (e.g., FIJI-ImageJ) will contribute to the reproducibility and usability of a study (D). Lastly, incorporating estimation statistics analysis to statistical analysis plan improves the interpretation of study outcomes by providing a quantitative measure of the extent of an outcome (i.e., effect size) and clearly depicting variability. The latter is further benefited by plotting all the data points and their respective distributions using highly descriptive types of scatterplots such as “*SuperPlots*” or Gardner-Altman and Cummings estimation plots (E). Implementing these items not only contributes to open science, but also enhanced the robustness of a research study.

Quantitative analysis of cell-types in mouse brain has enabled critical advances in understanding proper neurodevelopment and neurological disease in mammals (Stoner et al., 2014; DeTure and Dickson, 2019; Briscoe and Marín, 2020). However, the reusability of datasets and analysis methods is hampered by inconsistencies and errors in experimental design, gaps in the reporting of studies, as well as lack of user-friendly resources to design and produce systematic research studies that adhere to current best practices and open science principles (Button et al., 2013; Marqués et al., 2020; Picciotto, 2020). Given the growing (and much-needed) popularity of the open science movement (Koch and Jones, 2016), there is an impending need to elaborate and implement frameworks that are easy to adopt and adapt to each researcher interests. Here we provide an accessible and flexible framework for a commonly used study approach in neuroscience: quantification of cell density in a given brain region. Within this framework, we aimed to compile information that can help guide the implementation of open science workflows throughout the full span of an experimental research study, beginning with experimental design, tissue processing, and image acquisition, and concluding with an example of a workflow for image processing registration, and data analysis (Figure 4). This workflow takes advantage of the popularity, familiarity, and ease of use of the open-source platform for image analysis FIJI-ImageJ and availability of bio-image analysis community-built plugins, such as “*Bio-Formats*,” “*BigWarp*,” and “*StarDist*” (Linkert et al., 2010; Schindelin et al., 2012; Schmidt et al., 2018). We acknowledge that our proposed approach may not fulfill every researcher’s needs, and for this reason, we sought to complement our discussion with a compilation of emerging and in-development analysis tools (Supplementary Table 2). While simple, the flexibility of our framework allows for adaptation to capture more subtle or complex morphological features (e.g., quantifying cells with cytoskeletal markers) or to quantify other cell types (e.g., microglia, oligodendrocytes, and astrocytes) by modifying the image processing and segmentation steps with alternative FIJI-ImageJ-based tools and plugins. As the availability of bio-image analysis tools that use machine learning and artificial intelligence continues to grow, these tools can be implemented as modules within our proposed framework, expanding its capabilities and usability. We discuss some of these exciting new tools in Supplementary Table 2, such as those within the Python-based BrainGlobe platform (which includes “*brainreg*,” “*cellfinder*,” “*brainredner*,” and an “*ilastik*” implementation) which provide powerful and integrative analyses platforms for whole mouse brain sections, as well as alternative workflows to those published in the past (Berg et al., 2019; Yates et al., 2019; Claudi et al., 2021).

In all, the present work represents a compilation of key theoretical and practical considerations, and an operational framework that support and encourages a broader implementation of systematic and open-science workflows with the goal of creating greater synergy in our collective efforts as neuroscientists. Not only do we hope that this resource is helpful to the neuroscience research community, especially trainees, but we expect it will be further refined and improved upon.

## Data Availability Statement

The raw data has been deposited at the University of Victoria Scholar Portal Dataverse (https://doi.org/10.5683/SP2/KRGFTC). The scripts associated with this article are available on GitHub (https://github.com/SwayneLab/PFIA).

## Ethics Statement

The animal study was reviewed and approved by University of Victoria Animal Care Committee.

## Author Contributions

LAS designed the research together with JCS-A. Manuscript writing and revision was led by JCS-A, MC, and LAS. LAS, JCS-A, and SDF designed the image analysis workflow. SDF created the early versions of image analysis scripts with help from OS and PY for testing and development. JCS-A and HLN revised and refactored early versions of image analysis scripts. JCS-A redesigned the image analysis scripts and revised and refactored subsequent versions. JCS-A and EGA validated the redesigned image analysis workflow. OS prepared all brain tissues and performed the immunohistochemistry for fluorescent microscopy and image acquisition with help from PY. CM, OS, JCS-A, and SDF prepared the tables. LAS created Figures 1, 4. JCS-A created Figures 2, 3. JCS-A, LAS, EvdS, and MC revised all tables and figures. All authors contributed to the study, the writing, and revision of the manuscript.

## Conflict of Interest

The authors declare that the research was conducted in the absence of any commercial or financial relationships that could be construed as a potential conflict of interest.

## Publisher’s Note

All claims expressed in this article are solely those of the authors and do not necessarily represent those of their affiliated organizations, or those of the publisher, the editors and the reviewers. Any product that may be evaluated in this article, or claim that may be made by its manufacturer, is not guaranteed or endorsed by the publisher.

## Footnotes

1
https://www.stat.ubc.ca/rollin/stats/ssize/n2.html

2
http://powerandsamplesize.com/Calculators/

3
http://www.informatics.jax.org/

4
http://bioit2.irc.ugent.be/prx/me-pamufind-it/

5
https://scicrunch.org/resources

6
https://www.ibiology.org/online-biology-courses/microscopy-series/

7
https://github.com/SwayneLab/PFIA/blob/main/pfia_step_1_file_mgmt.ijm

8
https://huygens.science.uva.nl/ggPlotteR/

9
http://kimlab.io/brain-map/atlas/

10
https://github.com/SwayneLab/PFIA/blob/main/pfia_step_2_warp-register_brain_section.ijm

11
https://github.com/SwayneLab/PFIA/blob/main/pfia_step_3_count_pv_ cells.ijm

12
https://github.com/SwayneLab/PFIA/blob/main/pfia_step_3_count_neun_ cells.ijm
