# Supplementary material for: A Systematic, Open-Science Framework for Quantification of Cell-Types in Mouse Brain Sections Using Fluorescence Microscopy
Source: Front Neuroanat. 2021 Dec 6;15:722443. doi: 10.3389/fnana.2021.722443 (PMC8691181; doi:10.3389/fnana.2021.722443)
Supplement: Supplementary file 1 [file Data_Sheet_1.pdf]

Supplementary Methods

Experimental Animals.

All animal procedures were approved by the University of Victoria Animal Care Committee and performed in accordance with the guidelines set by the Canadian Council on Animal Care. Postnatal day 30-35 male and female C57BL/6J (#000664, The Jackson Laboratory) were used for immunohistochemistry experiments. Mice were housed under a 12/12 h light/dark cycle starting at 8 A.M., with food and water ad libitum; temperature was maintained between 20°C and 25°C and humidity at 40–65%. All mice were derived from harem breeding cages (2 females, 1 male) and weaned at postnatal day 21. The minimum number of weaned mice per cage was 2.

Preparation of tissue and immunohistochemistry

Mice were deeply anesthetized with isoflurane and transcardially perfused with phosphate buffer saline (PBS) followed by 4% paraformaldehyde, both at room temperature. Perfusion was performed with a pump to provide a constant flow of the fixative (flow rate: 8ml per min; duration: PBS, 2 min; 4% paraformaldehyde, 3 min). After decapitation, brains were dissected and furthered fixed in 4% PFA for 24h at 4°C, the dissection time was kept under 1 minute. Brains were numerically coded, and their identity was concealed from experimenter throughout immunohistochemistry, imaging, and cell analysis experiments. Free-floating coronal brain sections were obtained using a vibratome (Leica VT 1000S, Leica Microsystems, Germany) into 30 µm thick sections a speed of 0.5 mm/s and a vibration of 9 Hz and collected into 24-well culture dishes with PBS and stored at 4 °C until use. For immunohistochemistry experiments, free-floating brain sections within the anterior-posterior coordinates 0.26 mm and 0.5 mm were incubated with 10% normal donkey serum (017-000-121, Jackson ImmunoResearch) in PBS for 1 hour at room temperature, followed by primary antibody incubation using an antibody buffer (10% normal donkey serum and 0.3% Triton-X-100 in PBS) for 24 hours. After washing sections 3 times with PBS, secondary antibodies were incubated in antibody buffer for 1 hour at room temperature. Lastly, sections were washed 3 times in PBS and mounted with VectaShield (H-1000, Vector Labs) and a 1.5 borosilicate glass coverslip (22 x 22 mm, 50-365-603, Fisher-Scientific) and stored at -20°C until used for imaging experiments.

Primary and secondary antibodies

The primary antibodies used in the present study were: rabbit anti-NeuN (dilution, 1:500; Chemicon, Temecula, CA, USA), mouse anti-PV (dilution, 1:1000; Chemicon, Temecula, CA, USA). Secondary antibodies used in this study were: Alexa Fluor 488-conjugated AffiniPure donkey anti-rabbit IgG

(1:1000, 711-545-152), Alexa Fluor 594-conjugated AffiniPure donkey anti-mouse IgG (1:1000, 715-585-150). All secondary antibodies were obtained from Jackson ImmunoResearch. Optimal antibody dilutitions for immunofluorescence confocal microscopy were individually determined for each antibody using a serial dilution method.

Image acquisition

Sections were visualized under an inverted TCS Leica SP8 confocal microscope, equipped with a acoustic-optical beam splitter and the LASX Software Suite (version 3.1.3.16308). Three tile scans of the either left or right cerebral hemispheres were taken per mouse with using a 10× semi-apochromatic objective (HC PL FLUOTAR 10×/0.3; numerical aperture: 0.3, immersion: dry; individual size image size, 1024×1024; pixel size: 1.5 μm), and the following parameters acquisition parameters: pinhole, 4.0 AU; digital zoom, 0.75; frame average, 2; line accumulation; scanning speed: 600 Hz and bidirectional; z-step size, 2 μm; through the full thickness of the section (i.e., 30 μm). Images were acquired in sequential mode for each fluorophore using PMT detectors. The following laser lines and gain settings were used: for Hoechst, diode 405 at 0.7%, gain 737 mV; for AlexaFluor 488, Argon laser at 488 nm at 1.4%, gain 684 mV; for AlexaFluor 568, DPSS 561 nm at 2%, gain 661 mV. Optimal laser intensity, gain, offset, and pinhole settings were determined for each channel by setting the limits to include sub-saturated pixels and true-black pixels within a section. Images were collected to avoid pixel saturation. These settings were kept constant throughout image acquisition. Sections with poor perfusion, visible blood vessels and residual blood in the tissue were not further processed for imaging and analysis.

Image analysis

Image series in ".lif" files were processed in FIJI-ImageJ (initially v1.52c and then v1.53c). ImageJ Macro Language scripts were written within FIJI-ImageJ's text editor and tested on an image database of 5 male and 5 female C57BL/6J mice. Image series metadata was stored as ".csv" files and multi-channel image series were split into individual channels. The scrips associated with the image analysis workflow and test files are available at https://github.com/SwayneLab/PFIA and https://dataverse.scholarsportal.info/dataset.xhtml?persistentId=doi:10.5683/SP2/KRGFTC (doi: https://doi.org/10.5683/SP2/KRGFTC), respectively Both maximum projections and z-stack were stored, but analysis was only performed on 2D images. To compare the performance of manual tracing and a semi-automatic workflow, a junior research trainee and co-author and a senior research trainee and co-author were recruited. We selected randomly 5 multi-channel images series containing brain sections

immunolabelled for parvalbumin and NeuN. The barrel field cortex was isolated after registering each brain section to an outline from the unified mouse brain atlas generated by the Kim Lab (http://kimlab.io/brain-map/atlas/; (Chon et al., 2019)). Manual counts were done using the *"Multi Point"* tool on. Then, the senior research trainee analyzed the barrel cortex images using a conventional image thresholding and segmentation approach and *"StarDist"* (Schmidt et al., 2018; Weigert et al., 2020). The conventional approach consisted of was uniformly applying the thresholding method "*Moments*", (Tsai, 1985) to all images with parvalbumin-labelled cells, followed, by the binary operation "*Watershed*" and by size filtering and quantification using FIJI-ImageJ's "*Analyze Particle*" tool (size = 45 - 9000, circularity = 0.6 - 1.0). For NeuN-labelled cells, the *"Find Maxima"* tool (prominence = 15, exclude on edges) was used to quantify cell bodies.

Statistical analysis

Data was analyzed using R (R, Project, version 4.1.1) and RStudio (Version 1.4.1717, "Juliet Rose") and the following packages: readxl_1.3.1, tidyverse_1.3.1 (forcats_0.5.1, stringr_1.4.0, dplyr_1.0.7, purrr_0.3.4, tidyr_1.1.3, tibble_3.1.4, ggplot2_3.3.5), and magrittr_2.0.1. Comparison results were analyzed using a one-way ANOVA.

Supplemental Material

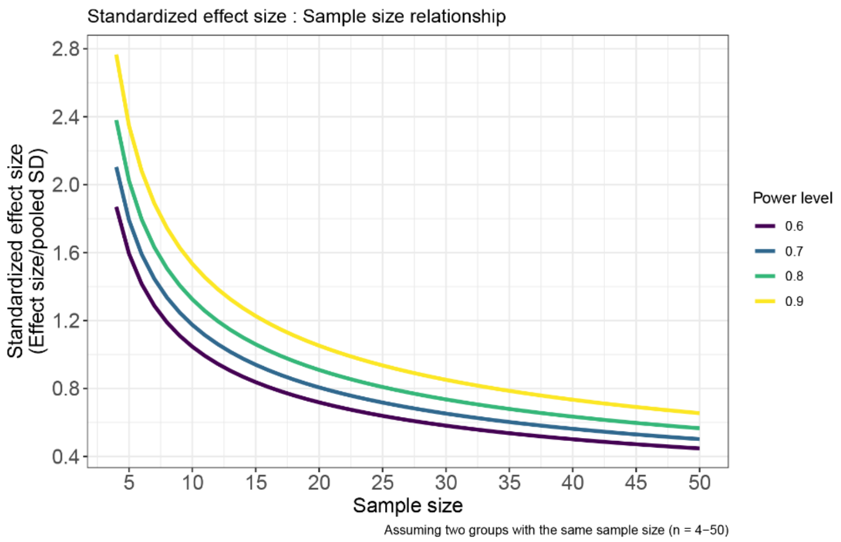

Figure 1. Relationship between standardized effect size and sample size. Simulation obtained by iterating the calculation of standardized effect size (difference of means/pooled standard deviation) of two groups for a given sample size (between 4 and 50) at various power levels (0.6 to 0.9). Classically, a power level of 0.8 (80%) or more is considered adequate.

Table 1. Histology quality control checklist			
Item / Procedure	Relevant information to report	Notes	References
Tissue processing	Use of fixative<br>- Type: e.g., aldehyde fixatives such as glyoxal, glutaraldehyde and paraformaldehyde (PFA) or alcohol, such as methanol<br>- Concentration.<br>- Perfusion speed and duration.<br>- Fixation solution temperature during fixation<br><br>Use of cryoprotective agents<br>- Type<br>- Concentration<br>- Incubation/treatment duration	4% Paraformaldehyde (PFA) in phosphate buffered saline is one of the most common fixatives used for immunofluorescence. The pH of 4% PFA in phosphate buffered saline should be around 7.4 to prevent the shrinkage or swelling of cells. Penetrates cells rapidly but has slower and weaker protein-cross linking ability than acrolein or glutaraldehyde. PFA is unable to crosslink lipids, and microtubules are not well preserved.<br><br>Acrolein reacts with fatty acids producing reversable crosslinks. Acrolein penetrates cells faster and deeper than PFA and glutaraldehyde. Can be difficult to work with due to formation of polymers and instability at alkaline pH.<br><br>Glyoxal provides relatively poor fixation compared to PFA. Glutaraldehyde fixation often reduces antigenicity and increases background autofluorescence. Rapidly fixes proteins but compared to PFA it has slow penetration into the cells.<br><br>Methanol fixation is typically used only when rapid fixation of cells is required. It is not recommended to use methanol fixation for immunofluorescence as it abolishes protein activity. Methanol is also known to cause shrinkage of the samples.<br><br>To prevent disruption to structure due to intracellular ice formation during freezing microtome or cryostat cutting, tissues should be infiltrated with cryoprotectants, such as sucrose, and frozen rapidly.<br><br>Air drying and fixation with aldehydes can cause distortion of tissues, affecting measurements.	Celikkan et al., 2020; Allan, 1999; Bacallao et al., 1995 ; Saito 1976
Slicing/Cutting method	Type and manufacturer (vibratome, rotary microtome, cryostat microtome)	Freezing microtome or cryostat tissue cutting is ideal for making thin frozen tissue sections for immunofluorescence. Disadvantages include tissue cracking and holes, fracturing of cell membrane, curled tissues, and intracellular ice crystal formation.<br><br>Vibratome cutting allows for preservation of ultrastructural features as it does not fracture cell membranes. Stains are sharper, with no cytosol in the extracellular tissue. Setup is rapid as tissues do not need to be embedded and can be directly glued to platform. Fragile samples can be embedded in agarose if necessary.	Paletzki, Gerfen. 2019

Permeabilization	Agent, concentration, incubation time	Permeabilization is required to create holes in plasma membrane (access for antibodies and fluorophores) if studying cytoplasmic proteins.<br><br>Triton® X-100 is a non-ionic detergent commonly used for permeabilization.<br><br>Saponins are natural compounds derived from plants, used for routine cytoplasmic antigen localizations. Saponin treatment produces smaller holes in membranes than Triton exposure.	North AJ. 2006; Melan, 1994
Labelling	Type (e.g., direct or indirect immunofluorescence)<br><br>Primary and secondary antibody information:<br>- Manufacturer (cat# and lot# if available)<br>- Additional manipulations<br>- Working concentration<br>- Clonality (polyclonal, monoclonal, recombinant)	Blocking solutions minimize unspecific binding and are normally used in concentrations of 1 % (bovine serum albumin) to 5 % (species-specific normal serum) and diluted in phosphate buffered saline. The incubation takes place at room temperature for 1 hour. To select the source species of a blocking agent, use the same species in which the secondary antibody was raised. Choosing a blocking agent from the same species in which the primary antibody was raised, will cause loss of the antibody's specificity.<br><br>Monoclonal antibodies offer single epitope specificity. They require higher working concentrations (5–25 mg/mL) compared to affinity-purified polyclonal antibody (typically ranging from 1.7–15 mg/mL). In general, polyclonal antibodies are also more stable than monoclonal antibodies over a range of pH and salt concentrations, making polyclonal antibodies very popular in immunofluorescence applications.	Lipman et al., 2005; Melan 1994
Mounting	Type and maker<br><br>Refractive index	The refractive index of the mounting medium should match the refractive index of the immersion agent for the objective and its numerical aperture. Mismatches in refractive indexes are an issue when imaging thick sections and tend to reduce resolution and hinder signal acquisition.<br><br>When possible, use a mounting medium that protects fluorophores from photobleaching. Confirm that your mounting medium is compatible with all the fluorophores in the sample, as reagents are not compatible with fluorescent proteins and will quench them (i.e., decrease the quantum yield).<br><br>Note that certain mounting media need curation and can lead to sample flattening or shrinkage of a sample (e.g., hardening mounting mediums). If avoiding sample shrinkage is a priority, use 50% glycerol in a buffered solution.	Bacallao et al., 1995; North, 2006

References

Allan, V.J. 1999. Basic immunofluorescence. In Protein Localization by Fluorescence Microscopy—A Practical Approach. V.J. Allan, editor. Oxford University Press, Oxford, UK. 1–26.

Bacallao, R., K. Kiai, and L. Jesaitis. 1995. Guiding principles of specimen preservation for confocal fluorescence microscopy. In Handbook of Biological Confocal Microscopy. 2nd edition. J.B. Pawley, editor. Plenum Press, New York. 311–325.

Celikkan FT, Mungan C, Sucu M, et al. PFA is superior to glyoxal in preserving oocyte, embryo, and stem cell proteins evidenced by super-resolution microscopical surveys of epitopes. *Journal of Assisted Reproduction and Genetics*. 2020 Feb;37(2):369-384.

Fouquet C, Gilles J-F, Heck N, Dos Santos M, Schwartzmann R, Cannaya V, et al. (2015) Improving Axial Resolution in Confocal Microscopy with New High Refractive Index Mounting Media. PLoS ONE 10(3): e0121096.

Paletzki R.F, Gerfen CR. Basic Neuroanatomical Methods. *Curr Protoc Neurosci*. 2019 Dec;90(1):e84.

Lipman N.S., Jackson L.R., Trudel L.J., Weis-Garcia F. 2005. Monoclonal Versus Polyclonal Antibodies: Distinguishing Characteristics, Applications, and Information Resources, *ILAR Journal*, Volume 46, Issue 3, 258–268

Melan M.A. (1994) Overview of Cell Fixation and Permeabilization. In: Javois L.C. (eds) Immunocytochemical Methods and Protocols. *Methods in Molecular Bilogy*, vol 34. Humana Press.

North AJ. Seeing is believing? A beginners' guide to practical pitfalls in image acquisition. *J Cell Biol*. 2006 Jan 2;172(1):9-18.

Saito T, Keino H. Acrolein as a fixative for enzyme cytochemistry. *J Histochem Cytochem*. 1976 Dec;24(12):1258-69. doi: 10.1177/24.12.187691. PMID: 187691.

Schmidt, U., Weigert, M., Broaddus, C., and Myers, G. (2018). Cell Detection with Star-Convex Polygons. in *Medical Image Computing and Computer Assisted Intervention – MICCAI 2018* Lecture Notes in Computer Science., eds. A. F. Frangi, J. A. Schnabel, C. Davatzikos, C. Alberola-López, and G. Fichtinger (Cham: Springer International Publishing), 265–273. doi:10.1007/978-3-030-00934-2_30.

Tsai, W.-H. (1985). Moment-preserving thresolding: A new approach. *Comput. Vis. Graph. Image Process.* 29, 377–393. doi:10.1016/0734-189X(85)90133-1.

Weigert, M., Schmidt, U., Haase, R., Sugawara, K., and Myers, G. (2020). Star-convex Polyhedra for 3D Object Detection and Segmentation in Microscopy. in *2020 IEEE Winter Conference on Applications of Computer Vision (WACV)*, 3655–3662. doi:10.1109/WACV45572.2020.9093435.

Supplemental Material

Table 2. Examples of image processing tools and workflows to quantify cell density in fluorescent and non-fluorescent labelled mouse brain sections

Name of analysis	*Context of previous use*	*Possible use*	*User Input*	*Program Output*	*Method Aspects*						*Reference*
					Objectivity	High efficiency	Customizability	User Friendly	Double staining	Compatibility with manual analysis	
QUINT workflow (combines the QuickNII tool, ilastik, and the Nutil tool)	Amyloid plaques in brain sections from transgenic mice and parvalbumin+ cells (*in situ*)	Adaptable for different cell and protein types in whole brain sections. Likely not adaptable for non-brain sections	- Ilastik component of workflow requires user to give label strokes to indicate object categories for segmentation	- QuickNII tool aligns serial section images to a 3D mouse brain reference atlas<br>- Ilastik performs image segmentation and classification of objects<br>- Nutil tool combines custom atlas maps and segmented images to quantify objects in specific brain regions and determine 3D object coordinates	✔	✔	✔	✔	✔	✔	Yates et al. (2019)
ilastik	3D neuron data, retinal images, colon cancer cells	Adaptable for different cell and tissue types and for 2D and 3D analysis	- User gives label strokes to indicate different object categories<br>- Fine tuning with additional labels until satisfied with classification result	- Live prediction mode after initial labels assigned<br>- Updated classification result after correction labels assigned<br>- After training with a few example images, it can predict classification on images of the same type	✔	✔	✔	✔	✔	✔	Sommer et al. (2011)
Brainglobe's Cellfinder and Brainreg	Coronal mouse brain section imaged using two-photon microscopy (*in vivo*)	Adaptable for different cell types in whole brain sections. Currently not adaptable for non-brain sections.	- User manually sets parameters for filters and thresholding	- Finds cell-like objects according to manually set parameters<br>- ResNet (deep-learning network) classifies cell-like objects as true cells or artifacts<br>- Brainreg allows for alignment of the image to a brain atlas with annotations allowing detected cells to be assigned to a brain region	✔	✔	✔	✗ ✔<br>*Documentation in development. Requires familiarity with python*	✔	NI	https://github.com/brainglobe/cellfinder
Cell Profiler	Drosophila Kc167 cells and human HT29 cells (*in situ*)	Highly adaptable for different cell and tissue types (can analyze any type of object). Not optimized for whole brain or brain hemi-sections.	- User selects modules to identify different components of imaged cells<br>- User adjusts settings for foreground versus background differentiation and for appropriate splitting and merging of objects	- Splits channels of multi-channel image<br>- Corrects for illumination<br>- Counts cell number according to adjustments made by user<br>- Can determine various other parameters including cell size, shape, and intensity	✔	✔	✔	✗ ✔<br>*Standalone software*	✔	✔	Carpenter et al. (2006)
Modified DAB-specific + pixel count algorithm	40 human tissues with 10 common antibody markers (*in situ*)	Adaptable for wide diversity of cell and tissue types	- Manually enter threshold values	- Designates pixels into negative, weak positive, positive, or strong positive bins	✔	✔	✔	✔	✗	✔	Chlipala et al. (2020)
SimplyCellCounter (SCC)	c-Fos+ cells in rat brain sections (*in situ*)	Adaptable for different cell and tissue types	- User sets threshold for radius size, pixel intensity, and circularity	- Binary mask to separate pixels above and below threshold<br>- Erosion and dilation steps to help remove background noise	✔	✔	✔	✔	✗	✔	Bal et al. (2020)

				- Hu moments to identify overlapping cells<br>- Pre-trained convolutional neural network to count cells							
Deep Learning and Unbiased Stereology	Neu-N+ neurons in mouse neocortex sections (*in situ)*	Likely adaptable for different cell and tissue types	- Method uses deep learning so minimal human intervention required.<br>- User must compare the adaptive segmentation algorithm to manual cell identification to see if they align	- Adaptive segmentation algorithm to segment stained cells<br>- Convolutional neural network to count the total number of cells using unbiased optical fractionator method	✔	✔	✔	✔	✗	✔	Alahmari et al. (2018)
Batch Counter plugin*	c-Fos+ cells in rat whole brain sections (*in situ*)	Adaptable for different atlases, landmarks, and ROIs	- Min. and max. cell size set manually in form of pixel range	- Plugin counts number and area of cells in specified brain regions	✔	✔	✔	✔	✗	✔	Bourgeois et al. (2021)
Automated Image Analysis with Background Subtraction***	Motor nuclei in *Drosophila* larval neuromuscular junction (*in situ*)	May be adaptable for different cell and tissue types	- Can use default parameters for motor neurons or manually set threshold range, cell size, and circularity	- Background fluorescence from outside ROI subtracted from each measurement<br>- Identifies motor nuclei and quantifies pixel intensity	✔	✔	✗	✔	✗	NI	Brown et al. (2019)

*****FIJI program used.; **ImageJ program used.; ***FIJI/ImageJ program used; NI: not investigated; NA: not applicable to method. In situ: imaging performed on tissues from an animal in an external environment with minimal alteration of natural conditions. In vivo: imaging performed in live animal, typically with two photon in-vivo microscopy.

References

Alahmari, S. S., Goldgof, D., Hall, L., Phoulady, H. A., Patel, R. H., & Mouton, P. R. (2019). Automated cell counts on tissue sections by deep learning and unbiased stereology. *Journal of chemical neuroanatomy*, *96*, 94-101.

Bal, A., Maureira, F., & Arguello, A. A. (2020). SimpylCellCounter: An automated solution for quantifying cells in brain tissue. *Scientific reports*, *10*(1), 1-10.

Bourgeois, J. R., Kalyanasundaram, G., Figueroa, C., Srinivasan, A., & Kopec, A. M. (2021). A semi-automated brain atlas-based analysis pipeline for c-Fos immunohistochemical data. *Journal of Neuroscience Methods*, *348*, 108982. https://doi.org/10.1016/j.jneumeth.2020.108982

Brown, J. R., Phongthachit, C., & Sulkowski, M. J. (2019). Immunofluorescence and image analysis pipeline for Drosophila motor neurons. *Biology Methods and Protocols*, *4*(1), 1–8. https://doi.org/10.1093/biomethods/bpz010

Carpenter, A. E., Jones, T. R., Lamprecht, M. R., Clarke, C., Kang, I. H., Friman, O., ... & Sabatini, D. M. (2006). CellProfiler: image analysis software for identifying and quantifying cell phenotypes. *Genome biology*, *7*(10), 1-11.

Claudi, F., Tyson, A. L., Petrucco, L., Margrie, T. W., Portugues, R., & Branco, T. (2021). Visualizing anatomically registered data with brainrender. ELife, 10, e65751. https://doi.org/10.7554/eLife.65751

Chlipala, E. A., Bendzinski, C. M., Dorner, C., Sartan, R., Copeland, K., Pearce, R., Doherty, F., & Bolon, B. (2020). An Image Analysis Solution for Quantification and Determination of Immunohistochemistry Staining Reproducibility. *Applied Immunohistochemistry and Molecular Morphology*, *28*(6), 428–436. https://doi.org/10.1097/PAI.0000000000000776

Ibanez, F. G., Picard, K., Bordelau, M., Sharma, K., Bisht, K., & Tremblay, M. È. (2019). Immunofluorescence staining using iba1 and tmem119 for microglial density, morphology and peripheral myeloid cell infiltration analysis in mouse brain. *Journal of Visualized Experiments*, *2019*(152), 1–8. https://doi.org/10.3791/60510

Rogalla, M. M., & Hildebrandt, K. J. (2020). Aging but not age-related hearing loss dominates the decrease of parvalbumin immunoreactivity in the primary auditory cortex of mice. *Eneuro*, *7*(3).

Sommer, C., Straehle, C., Koethe, U., & Hamprecht, F. A. (2011, March). Ilastik: Interactive learning and segmentation toolkit. In *2011 IEEE international symposium on biomedical imaging: From nano to macro* (pp. 230-233). IEEE.

Woeffler-Maucler, C., Beghin, A., Ressnikoff, D., Bezin, L., & Marinesco, S. (2014). Automated immunohistochemical method to quantify neuronal density in brain sections: application to neuronal loss after status epilepticus. *Journal of neuroscience methods*, *225*, 32-41.

Yates, S. C., Groeneboom, N. E., Coello, C., Lichtenthaler, S. F., Kuhn, P. H., Demuth, H. U., ... & Bjaalie, J. G. (2019). QUINT: workflow for quantification and spatial analysis of features in histological images from rodent brain. *Frontiers in neuroinformatics*, *13*, 75.
